# Supplementary material for: Host Specificity of the Parasitic Wasp Anaphes flavipes (Hymenoptera: Mymaridae) and a New Defence in Its Hosts (Coleoptera: Chrysomelidae: Oulema spp.)
Source: Insects. 2020 Mar 10;11(3):175. doi: 10.3390/insects11030175 (PMC7143892; doi:10.3390/insects11030175)
Supplement: Supplementary file 1 [file insects-11-00175-s001.zip › Supplementary_Materials/Supplementary_Material_3.pdf]

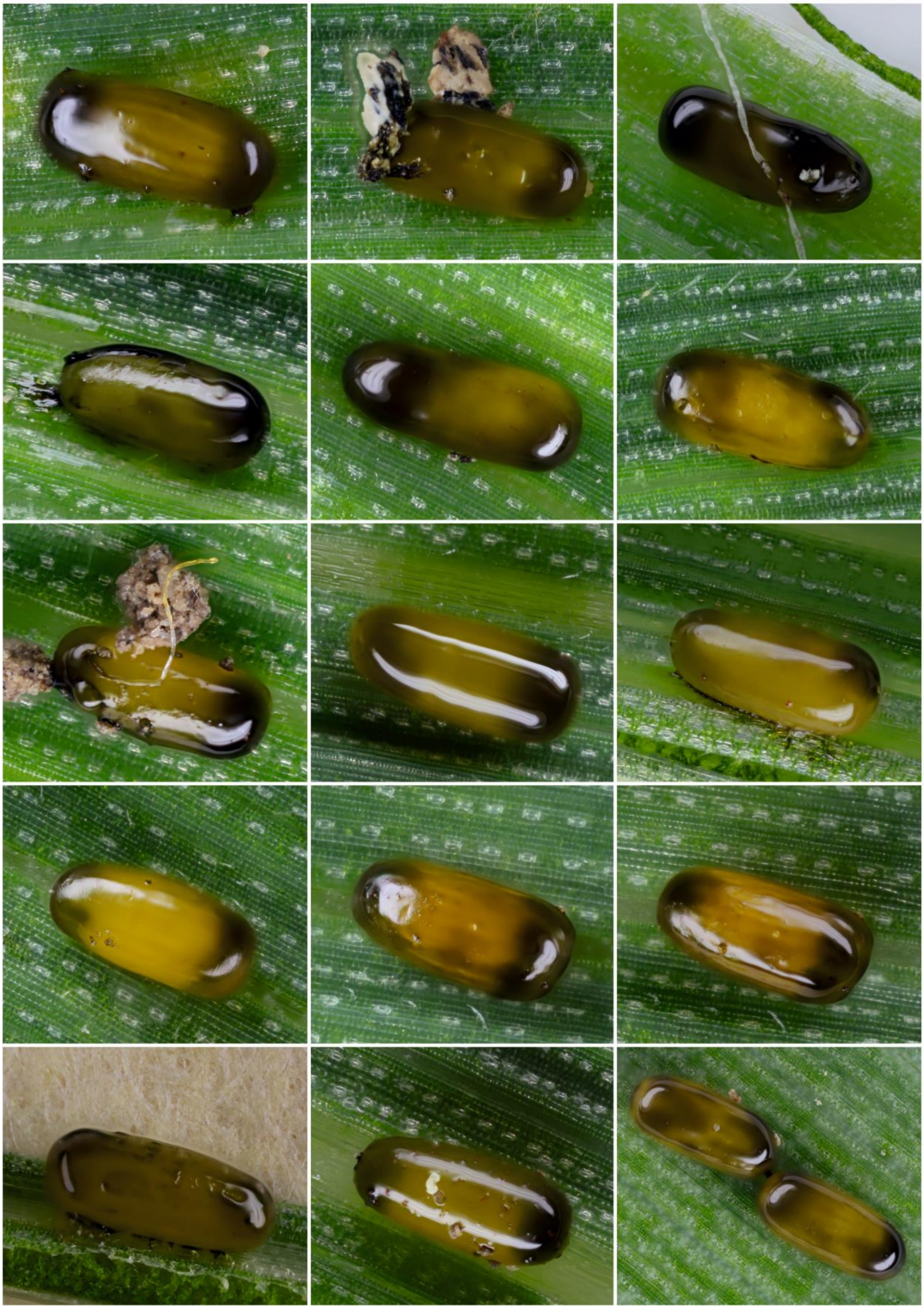

Supplementary Material 3. – The eggs with dark sticky layer (*Oulema gallaeciana* Heyden, 1879 from Czech Republic).
